# 

**Published:** 1916-05

**Authors:** 


### CONTENTS, MAY, 1916—VOLUME XXXI, NO. 11.

ORIGINAL ARTICLES.

The Treatment of Neuralgia by Alcoholic and Other Injections. By Wilse Robinson, M. D., Kansas City, Mo...... 441

Diagnosis of the Internal Secretory Disorders. By Henry R. Harrower, M. D., F. R. S. M. (Lond.), Los Angeles, Cal.. 449

Malignant Disease of the Skin. By Dr. W. L. Crosthwait, Waco, Texas ........................................ 454

Old Fashioned Drugs. By Dr. G. Henry Bogart, Shelbyville, Ill. ........................................ 460

Just Some Observations on Childhood. By E. S. Goodhue, A. M., M. D., LL. D., Hawaii........................ 462

EDITORIAL DEPARTMENT.

The Cost of Disease........................................ 468

They Show Progress........................................ 468

Fighting Diseases ........................................ 469

Pellagra Preventable ........................................ 469

County Hospitals ........................................ 470

A Valuable Series........................................ 470

This Journal is Yours........................................ 470

Abstracts and Selections........................................ 471

Items of General Interest........................................ 474

Personal News Items........................................ 477

Change of Location........................................ 478

Deaths ........................................ 480

Book Reviews ........................................ 480

Helpful Hints for the Busy Doctor........................ 482

